# Prevalence of latent tuberculosis infection among health care workers in a hospital for pulmonary diseases

**DOI:** 10.1186/1745-6673-4-1

**Published:** 2009-01-09

**Authors:** Anja Schablon, Gudrun Beckmann, Melanie Harling, Roland Diel, Albert Nienhaus

**Affiliations:** 1 Institution for Statutory Accident Insurance and Prevention in the Health and Welfare Services, Department of Occupational Health Research, Pappelallee 35-37, 22089 Hamburg, Germany; 2 Hospital of Pulmonary Diseases Großhansdorf Hamburg, Germany; 3 School of Pulbic Health, Institute of Medical Sociology, University of Düsseldorf, Germany

## Abstract

**Background:**

Little is known about the prevalence of latent tuberculosis infections (LTBI) in health care workers (HCW) in low-incidence countries especially in hospitals for pulmonary diseases. With Interferon-gamma release assays (IGRA), a new method for diagnosis of LTBI is available which is more specific than the tuberculin skin test (TST).

**Objectives:**

The study was designed to estimate prevalence of LTBI among 270 HCW in a Hospital of Pulmonary Diseases routinely screened for TB.

**Methods:**

LTBI was assessed by the QuantiFERON-Gold In Tube (QFT-IT). Information on gender, age, workplace, job title, BCG vaccination and history of both TB and TST were collected using a standardised questionnaire. Adjusted odds ratios for potential risk factors for LTBI were calculated.

**Results:**

The prevalence of LTBI was 7.2%. In HCW younger than 30 years LTBI prevalence was 3.5% and in those older than 50 years 22%. Physicians and nurses showed a higher prevalence rate than other professions (10.8% to 4.5%). The putative risk factors for LTBI were age (>50 year OR 9.3, 95%CI 2.5–33.7), working as physicians/nurses (OR 3. 95%CI 1.2–10.4) and no previous TST in medical history (OR 4.4, 95%CI 1.01–18.9) when compared to those with a negative TST.

**Conclusion:**

Prevalence of LTBI assessed by QFT-IT is low, this indicates a low infection risk even in hospitals for pulmonary diseases. No statement can be made regarding the occupational risk as compared to the general population because there are no LTBI prevalence data from Germany available. The higher LTBI prevalence rate in older HCWs might be due to the cohort effect or the longer time at risk.

## Background

Germany is a country that developed from a high tuberculosis incidence country to a low incidence country during the last 50 years. Since the 1950s, the number of new tuberculosis (TB) cases in Germany decreased from 9.064 newly diagnosed TB cases in the year 2000 (Federal Department of Statistics, 2000) to 5.402 in 2006 [1].

The introduction of effective control and preventive measures against tuberculosis transmission, the advent of an effective treatment for tuberculosis and the concurrent long-term downwards trend of tuberculosis incidence substantially decreased the occupational risk among healthcare workers [2]. Currently, in industrialised countries such as Germany, occupational tuberculosis among HCWs is re-emerging as an important public health issue, because of the resurgence of tuberculosis epidemic in former Soviet Union (NIS) states, the emergence of multidrug-resistant strains of mycobacteria especially in these countries and increasing migration from exactly these countries [3]. Furthermore, the occurrence of TB as a co-infection to HIV especially in the US has resulted in a flare-up of the discussions about this work-related infection risk and in the initiating of a large number of related epidemiological studies [4]. In addition, molecular epidemiological studies have shown that even in countries with a low TB incidence, 30 to 40% of all cases are cases of "newly transmitted TB" [5-7].

In high-income countries (HIC) relatively few LTBI prevalence surveys have been published since 1990. Findings were consistent with earlier surveys in that the median prevalence of positive TST was 24% with a range from 4% to 46 [8]. So far, these conventional studies on prevalence of LTBI in HCW used the TST and were thus hampered by the low specificity of the TST and its cross-reactivity with BCG and nontuberculous mycobacteria (NTM) infections [9].

The *M. tuberculosis*-specific interferon-gamma (IFN-γ) -based diagnostic tests may improve this situation [10-12]. Two IGRAs, Quantiferon-TB and the T-spot TB are now commercially available. The third generation of the Quantiferon test (Quantiferon^®^TB Gold In-Tube, QFT-IT) measures *in vitro *IFN-γ production by the T-cells during *in vitro *stimulation with peptides of the *M. tuberculosis*-specific antigens of the region of difference (RD-1) ESAT-6, CFP-10 and TB7.7. These antigens are not shared by any of the BCG vaccine strains nor by the more common species of NTM (e.g. *M. avium*) [13-15]. Available evidence reviewed elsewhere [12,13,16] suggests that these Interferon-γ release assays have higher specificity and at least equal sensitivity as the TST and are unaffected by previous BCG vaccination and most NTM. Therefore this test reduces the risk of LTBI overestimation due to cross-reactions with BCG vaccination or exposure to environmental mycobacteria [16].

So far only few studies have investigated the prevalence of LTBI in HCWs in low-incidence countries with the new *in-vitro *tests [17-22]. These prevalence rates are much lower than those assumed for German HCW so far [23].

Employees in hospitals for pulmonary diseases are among those individuals who are routinely screened for TB as stipulated by German OSH legislation [24]. It is assumed that this occupational field bears an increased risk of M. tuberculosis infection for the employees because their institutions frequently treat TB patients [8].

Out of 247 hospital workers of a German Hospital for Pulmonary Diseases in Großhansdorf on average 1 TB case per year occurred in the 30 years from 1950 to 1979. Most TB-cases appeared at medically technical professions (31.3%) followed by doctors (23%), nurses (13.6%) and other non medical professions. While for the general German population a sharp decrease in TB incidence was observed in this time period there was no significant decrease in TB incidence in the hospital staff. According to the authors this indicated a "strong, flowing source of infection" [25]. This hospital is still the referral center for TB treatment in Hamburg. Therefore we analyzed the prevalence of LTBI in the staff of this hospital with the QFT-IT in order to assess the strength of the "source of infection" more than 25 years later.

## Methods

### Study design

We conducted a cross-sectional study in a hospital for pulmonary disease in the northern part of Germany. The clinic has three wards specialized in pneumology, pneumology/oncology, and thoracic surgery, with a total capacity of 213 beds. About 8,500 in-patients and 4,500 out-patients from all over Northern Germany are treated each year. The clinic has 350 staff members (40 physicians, 150 nurses, 80 employees in the areas of radiology, pulmonary function, laboratory etc. plus 20 in anesthesia/surgery. One of the treatment foci is tuberculosis. 60 TB patients are treated per year, 95% of them presenting with infectious pulmonary TB. Up to 10% of these TB-cases were drug resistant, particularly MDR, and up to 3 cases were multi drug resistant (XDR). 75% of the TB patients treated per year are referred without a diagnosis or suspicion of TB. Only 25% of the patients were already diagnosed or referred with the suspicion of TB. These patients were isolated on arrival. The clinic has a special TB-ward but no engineering controls such as ventilation and UV light. The study population consisted of HCWs tested between December 2005 and January 2008, either in the course of a contact tracing or in serial testing of TB high-risk groups following German OSH legislation (Biostoffverordnung). A total of 270 HCW were enrolled in the study. There were no exclusion criteria for study participants.

### Diagnostic methods

For the IGRA, the QuantiFERON-TB Gold In-Tube test was used (Cellestis Limited, Carnegie, Australia). This whole-blood assay uses overlapping peptides corresponding to ESAT-6, CFP-10, and a portion of tuberculosis antigen TB7.7 (Rv2654). Stimulation of the antigenic mixture occurs within the tube used to collect the blood. Tubes were incubated at 37°C overnight before centrifugation, and INF-γ release was measured by ELISA following the protocol of the manufacturer. All the assays performed met the manufacturer's quality control standards. The test was considered positive if INF-γ was ≥ 0.35 UI/ml after correction for the negative control.

### Questionnaire items

Information on the following variables was collected using a standardized questionnaire: age, gender, reason for testing, occupational exposure to TB, time of occupation in health care sector, reason for testing, family history of TB, BCG vaccination, place of birth, prior TST, job title, workplace and chest radiographic findings. BCG vaccination was verified by scars or vaccination records.

The study protocol was approved by the ethics committee of the Hamburg Medical Council. All persons gave their written informed consent prior to their inclusion in the study.

### Statistical analysis

Data analysis was performed using SPSS, Version 14 (SPSS Inc., Chicago, Illinois). The study population comprises 270 HCWs, which means that more than two thirds of the hospital staff were examined. Due to indeterminate QFT-IT result 5 HCWs (1.9%) were excluded from the analysis. Adjusted Odds ratios for QFT-IT depending on different putative predictive variables were calculated using logistic regression. Model building was performed backwards using the chance criteria for variable selection [26].

## Results

The mean age of the participants was 34.7 ± 12.6 years. The majority of the participating HCWs (74%) were female and the mean age was 34.7 years (SD ± 12.6). A history of BCG vaccination was recorded for 52.8% of the participants. 19.2% of the study population were born outside Germany or had a history of migration and 80.2% of the study population were born in Germany (table 1). Most of the foreign-born participants came from Turkey, former Soviet Union (NIS) states and Eastern Europe, e.g. Poland and Bulgaria. 25.5% of the employees with a history of migration were physicians or nurses. The vast majority worked in non-medical areas, for example as cleaners (56.9%). None of the foreign-born physicians/nurses was QFT-IT positive (no table).

**Table 1:** Description of the study population

**Variable**	**N**	**%**
Gender		
Female	196	74
Male	69	26

Age*		
< 30 years	115	43.4
30 – < 40 years	41	15.5
40 – < 50 years	68	25.7
50 – 67 years	41	15.5

Country of birth		
Germany	214	80.8
foreign country	51	19.2

BCG-vaccination		
No	125	47.2
Yes	140	52.8

TST history		
No TST in medical history	33	12.5
Negative TST in history	151	57
Positive TST in history	81	30.6

Job category		
Nurse	94	35.5
Physician	17	6.4
Other professions	154	58.1

QFT-IT		
Negative	246	92.8
Positive	19	7.2

Workplace		
Admission ward	18	6.8
Infection ward	110	41.5
Other	137	51.7

Reason for testing		
Serial examination	246	92,8
Contact tracing	19	7,2

Total	265	100

* mean age 34.7, standard deviation 12.6

35.5% of the total study population were nurses, 6.4% physicans and 58.1% were other professions in health care sector or non-medical staff including cleaners, transportation service staff, physiotherapists, interns, radiology staff, conscientious objectors, apprentices and administrative staff. In the subgroup other professions only two persons with direct patient contact (one physiotherapist and one radiology staff member) are included. These two persons were positive by QFT-IT but either 5 cleaners and administrative staff members were QFT-IT positive (data not shown). 92,8% (n = 246) of the HWCs were investigated because of serial examinations of high risk groups following the German OSH legislation (Biostoffverordnung) and 19 (7,2%) of these participants were positive by QFT-IT. 19 HCWs were investigated in course of contract tracing but nobody in these group was positive by QFT-IT (table 1).

A positive QFT-IT result was observed in 19 (7.2%) participants and 81 participants reported a positive previous TST (table 1). Out of 33 participants with no TST in their medical history, 4 persons (12,1%) were now positive by QFT-IT (TB-antigen-Nil range from 0,44 to 1,50). Of the 81 participants positive in a previous TST, 10 (12.3%) were confirmed by the IGRA (TB-antigen-Nil range from 0,46 to 270,2)(no table).

The prevalence of LTBI assessed by QFT-IT correlated with age. In the subgroup with participants under 30 years old, LTBI prevalence was 3.5%. In the subgroup with participants between 50–67 years the prevalence increased to 22%. LTBI prevalence was higher in physicians and nurses (10.8%) than in other areas of occupation (4,5%) within the hospital. In the wards of pulmonology/Infectious diseases, QFT-IT was less often positive than in the admission ward (3.6 versus 11%). However, the differences were not statistically significant (table 1).

The putative risk factors for a positive QFT-IT were age (>50 year OR 7.7, 95%CI 2.1–28.2) and working as a physician/nurse (OR 3.2, 95%CI 1.1–9.0). Using the subgroup with a negative TST in history as comparison group, the odds ratio for those with no previous TST in medical history was elevated (OR 4.4, 95%CI 1.01–18.9). The latter statistically significant association was observed after adjustment for age and job category, it did not show in the crude data. No statistically significant association was observed for gender, BCG vaccination, workplace and migration (Table 2).

**Table 2:** Frequency and Adjusted Odds ratios (OR) and 95% Confidence Interval (95%CI) for Covariates associated with QFT-IT Results.

	**QFT-IT**			
Characteristics	negative N (%)	positive N (%)	Odds Ratio	95% CI

Gender**				

female	182 (92.9%)	14 (7.1)	1	
male	64 (92.8)	5 (7.2)	1.3	0.4–4.2

Age*				

>30 years	111 (96.5)	4 (3.5)	1	
30–40 years	39 (95.1)	2 (4.9)	1.2	0.2–7.0
40–50 years	64 (94.1)	4 (5.9)	1.5	0.4–6.5
50–67 years	32 (78.0)	9 (22.0)	**7.7**	**2.1–28.2**

Country of birth**				

Germany	198 (92.5)	16 (7.5)	1	
foreign born	48 (94.1)	3 (5.9)	0.9	0,2–3,3

Job Category*				

other professions	147 (95.5)	7 (4.5)	1	
physician/nurse	99 (89.2)	12 (10.8)	3.2*	**1.1–9.0**

BCG vaccination**				

no	114 (91.2)	11 (8.8)	1	
yes	132 (94.3)	8 (5.7)	0.5	0.2–1.3

TST history*				

negative TST in history	146 (96.7)	5 (3.3)	1	
no TST in medical history	29 (87.9)	4 (12.1)	**4.4**	**1.01–18.9**
positive TST in history	71 (87.7)	10 (12.3)	3.0	0.95–9.6

Workplace**				

other	124 (90.5)	13 (9.5)	1	
admission ward	16 (88.9)	2 (11.1)	1,4	0.3–79.2
infection ward/pulmology	106 (96.4)	4 (3.6)	0,4	0.1–1.3

* the final multivariate logistic regression model contains the variable age, job category and TST history.

** adjusted Odds ratio for age, job category and TST history.

All participants with a positive QFT-IT were offered a clinical and radiologic examination to rule out active TB. None of them showed any clinical or radiological sign of active TB disease and hence no further action was taken.

## Discussion

In this study, we have found that the prevalence of LTBI assessed by QFT-IT is low and it is considerably lower than assumed in the past [23]. With the IGRA we have, for the first time, a test that allows for valid statements regarding the LTBI prevalence, infection risk and disease probability.

Only few studies in low-incidence countries have so far employed the IGRA as a screening instrument in health care workers [17-22]. In accordance with the literature, Nienhaus et al. investigated 261 HCWs from different types of hospitals who are routinely screened for TB as stipulated by German OSH legislation using QFT-IT and TST following the German Guidelines with a cut off >5 mm. LTBI prevalence assessed by QFT-IT was 9.6% compared to 24.1% with TST [17]. Furthermore, Soborg and colleagues used QFT-Gold TB to test 139 HCWs at two departments for infectious diseases in Copenhagen. 105 HCW had direct patients contact and 34 HCW were employed with office work and had no daily patient contact. They found an LTBI prevalence rate of only 1% (n = 2) as compared to 34% (n = 47) with the TST (cut off >12 mm); and this rate was much lower than the estimated prevalence (7.2%) in our study [18]. Stebler et al. also studied the prevalence of LTBI among hospital employees at the University Hospital of Berne using the IGRA. A total of 777 HCWs were investigated. A positive IGRA was found for 59 (7,6%) [22]. In addition Harada et al. investigated the performance of the QFT-G for detecting LTBI by testing 332 HCWs in a Japanese general hospital and suggested a prevalence of LTBI of 9.9% [19]. Kobashi and colleagues found a prevalence of LTBI of 3% among 109 HCWs who were examined during contact investigations [20]. In the study among 95 HCWs working in departments of radiology, Barsegian et al. observed a prevalence of LTBI of 1% using the T-SPOT in 95 German radiologists [21].

The relatively low rate of positive QFT-IT we found in HCWs in a German hospital for pulmonary diseases indicates a low infection risk even in this occupational area. Especially in working fields with an increased risk of TB exposure, effective control measures are an important tool to reduce TB transmission. Because TB treatment is one of the focus areas of hospitals for pulmonary diseases, it can be assumed that physicians and nurses are familiar with the appropriate protective measures. As patients are referred to the clinic after some clinical evaluation elsewhere, TB cases may be identified early or even before referral and effective control measures may be taken.

In this study, we found no indication for a "strong, flowing source of infection". From 1950 to 1979 a total of 29 employees of the Pulmonary Hospital of Großhansdorf developed active TB while the overall TB incidence in the general population was decreasing. The authors concluded that the staff of this hospital was exposed to a greater risk than the population of Germany or other industrialized countries [25]. In the scope of the present examination, none of the employees developed active TB in the observation time of 1 year. Only 7.2% of the employees were QFT-IT positive. This may indicate further improvement of the TB control measures.

In contrast to our findings, three molecular biological studies have found a job-related exposure to TB for HCWs. First, a molecular biological fingerprint study from Hamburg/Germany (n = 848) has shown that the risk of active TB for HCWs is not increased as compared to the general population. However when disease occurred, the infection is most probably due to job-related exposure. In the Hamburg fingerprint study, a total of 10 HCWs developed TB; a job-related infection was established in 8 of them (80%) [5]. Second, Ong et al. [6] failed to detect an increased TB rate among HCWs in San Francisco in their fingerprint study (n = 2510). The proportion of clustered cases in HCWs (32%) was similar to that observed in the community (36%). In at least 10 (32%) of the HCWs, there was genotyping and/or epidemiogical evidence of job-related transmission [6]. Third, the objective of the epidemiological and microbiological study of de Vries et al. was to determine which TB cases among HCWs in the Netherlands were infected during work. Of a total of 101 TB cases, the infection pathways of 67 cases could be established; 42% (28 out of 67) were due to infection at work [7].

In our study a positive QFT-IT result was associated with age (>30 years 3.5%, 50–67 years 22%), no previous TST in the medical history and occupation as a physician or nurse. The higher prevalence rate in older HCWs might be due to a cohort effect or the longer time at risk. HCWs without a previous TST in their history had an increased OR compared to those with a negative TST in history even after controlling for risk of infection. A clear explanation to this was not found. Probably those with a negative TST once in history might be genetically protected against infection. HCWs with an earlier positive TST also had an increased OR which was, however, not statistically significant. This may be due to the small size of the group. A booster phenomenon when IGRA are applied after TST can be excluded because the QFT-IT was carried out in front of the TST.

In the recent review from Menzies et al. [8] occupational risk factors were associated with work in internal or respiratory medicine, years of work in healthcare, more direct indicators of TB exposure, including TB admission or the percentage of patients with TB or HIV cared for [8]. Another reason for transmission in healthcare settings was the delayed diagnosis of the index case, especially in elderly patients. In the HCW study by De Vries et al. 44% of the index patients were older than 60 years. Delayed diagnosis in older patients was the main cause of patient-to-HCW transmission in the Netherlands [7].

So far the occupational LTBI infection risk of nurses has been investigated in several conventional studies. Most studies showed a statistically significant increase of >2 in relative risk [27]. The infection risk of physicians was examined in various studies of varying quality. Because of the inadequate data, it is difficult to evaluate the tuberculosis risk in different medical specialties. The results are contradictory and all in all do not indicate an increased infection risk for physicians. Pathologists have an increased risk of infection but no pathologist were included in this study. Several studies also identified a statistically clearly increased TB infection risk of the staff of wards where TB patients are treated; thus the increased infection risk of HCWs in areas with TB patients is epidemiologically ascertained [27].

The IGRAs proves to be a more important screening instrument for LTBI diagnosis in low-incidence countries as it allows valid statements on the prevalence and incidence of LTBI. The IGRA can help to identify at-risk groups and reduce the indication of preventive chemotherapy. The use of IGRAs in serial testing of health care workers is still not very well studied and the influence of the role of potential dynamic of IGRA responses still needs to be clarified [28]. Most of the serial testing studies have been done in high-incidence countries and show inconsistent results. Very few studies so far have been done on disease progression [29,30]. The data indicate that a positive IGRA correlate with a high progression rate but the number of cases is still small and these results need to be interpreted with care. The recent study from Diel et al. (2008) on disease probability after positive IGRA showed that, out of 41 participants with a positive IGRA result, 14.6% developed TB within the 103 weeks of observation. The progression rate for TST-positives was only 2.3% [29]. Thus the progression rate estimated by IGRA was higher than the one estimated by WHO [31] for lifetime after positive TST (5 to 10%).

## Limitations

As changes with time cannot be considered in a cross-sectional study, the OR can provide evidence for factors influencing the results, but only restricted conclusions about the causality of these correlations are possible. To allow for a comparison between professions under risk we created a variable with the groups Nurse, Physician and other professions (reference group). It was known, that in the unexposed group might also be employees with contact to patients (e.g. physiotherapist and radiology staff), but there was only two case of QFT-IT positive. This may limit the generalizability of our results.

## Conclusion

In summary, the prevalence of LTBI assessed by QFT-IT in a hospital for pulmonary diseases is rather low. Other than in the years before 1980, we found no indication for a "strong, flowing source of infection". No statement can be made regarding the occupational risk as compared to the general population because there are no data from Germany available for comparison. It is important, especially in high-risk settings, to follow the current guidelines for the prevention of tuberculosis in the workplace, including appropriate patient risk assessment, active hospital tuberculosis case surveillance and development of an effective institutional infection control plan to reduce the transmission rate of tuberculosis in healthcare settings [6-8]. Disease probability in HCWs tested positive by serial testing should be investigated in longitudinal studies

## Competing interests

The authors declare that they have no competing interests.

## Authors' contributions

GB has made substantial contributions to acquisition of data. She has been involved in revising it critically for important intellectual content.

RD has made substantial contributions to conception and design of the study. He has been involved in revising the manuscript critically for important intellectual content,

MH has made substantial contributions to interpretation of data. She has been involved in revising the manuscript critically for important intellectual content.

AN has made substantial contributions to conception and design, as well as to analysis and interpretation of data. He has been involved in drafting the manuscript

AS has made substantial contributions to conception and design, acquisition of data, as well as to analysis and interpretation of data. She has been involved in drafting the manuscript
